# Isolation and Classification of Fungal Whitefly Entomopathogens from Soils of Qinghai-Tibet Plateau and Gansu Corridor in China

**DOI:** 10.1371/journal.pone.0156087

**Published:** 2016-05-26

**Authors:** Tingyan Dong, Bowen Zhang, Yanfang Jiang, Qiongbo Hu

**Affiliations:** Key Laboratory of Bio-Pesticide Innovation and Application of Guangdong Province, College of Agriculture, South China Agricultural University, Guangzhou, 510642, China; Zhejiang University, CHINA

## Abstract

Qinghai-Tibet Plateau and Gansu Corridor of China with distinct geographic and climatic conditions are remote and less disturbed by humans, in which are likely to find some new strains of fungal entomopathogens against B-biotype whiteflies that is a very important invading pest worldwide. In this research, nineteen strains among six species of entomogenous fungi were isolated from the soil samples collected from 32 locations in Qinghai-Tibet Plateau and Gansu Corridor. From the data of isolation rates, it was indicated that the good biodiversity of entomogenous fungi was found in the soil covered good vegetations. On the contrary, no strains were isolated from the desert areas. In addition, the dominant species, *Isaria fumosorosea* and *Metarhizium anisopliae* var. *anisopliae* in the Qinghai-Tibet Plateau are different from the strains of other places based on ITS genetic homology analysis. It was verified that the Qinghai-Tibet Plateau area was less disturbed by human, and the fungi in this place exchanged less compared with other regional species. All of these strains showed the pathogenicity against the B-biotype whitefly with the mortality of more than 30%. However, a few strains of *Paecilomyces lilacinus*, *Lecanicillium psalliotae*, *Aspergillus ustus*, *I*. *fumosorosea* and *M*. *anisopliae* var. *anisopliae* had better virulence with LC_50_s of 0.36–26.44×10^6^ spores/mL on post-treatment day 6–7. Especially, the *L*. *psalliotae* strain LpTS01 was the greatest virulence with LC_50_ of 0.36×10^6^spores/mL and LT_50_ of 4.23d. Our research thus presents some new insights to discover new entomopathogenic fungal strains used for B-biotype whitefly biocontrol.

## Introduction

The whitefly, *Bemisia tabaci* (Gennadius) (Hemiptera: Aleyrodidae), is a complex insect species including many cryptic species or biotypes. The two biotypes of *B*. *tabaci*, biotype B and biotype Q are serious invading pests in the world. They harmfully influence the production of horticultural crops by directly feeding on phloem sap, resulting in sooty mould and seriously reducing the photosynthesis of host plants. They also indirectly damage crop production by transmitting begomoviruses diseases such as tomato yellow leaf curl virus (TYLCV) [1–3]. In China, the B- biotype whitefly was first recognized in the late 1990s, after which it rapidly spreads all over the country with the exception of Tibet [4]. The Q-biotype whitefly was found in the early 21st century and was considered to be a substituent of the B-biotype [5]. In recent years, chemical insecticides have been massively used to control *B*. *tabaci*. Accordingly, the pesticide resistance and residues in foods are attracting public attention. The biocontrol technology is necessary for integrated pest management (IPM) of whitefly.

Entomogenous fungi infect insects and lead to epidemic disease in proper conditions. Many of fungal entomopathogens have been developed as mycoinsecticide. The fungal agents with advantages of non-resistance and non-contamination are considered as an alternative means for the control of whitefly, and thus, it is attracting more public and scientific interests [6–8]. Entomopathogenic fungi directly invade host insects through penetrating the cuticles, so that benefit to the management of piercing–sucking pests. To date, more than 20 species of entomopathogenic fungi have been known to infect whiteflies. Among these, *Isaria fumosorosea* (*Paecilomyces fumosoroseus*), *Verticillium lecanii* and *Beauveria bassiana* have had been most widely studied [9–12]. The formulation and application of entomopathogenic fungi based on these fungal entomopathogens have been being used to control whitefly populations in both greenhouse and field crops [13–14]. Nevertheless, numerous factors, such as slow action, unstable effectiveness and limited shelf life are known to hinder the development of mycoinsecticids. Therefore, there is an increasing demand for the investigation of potential fungal strains with a higher virulence and resisting performance against pests.

Soil is an important reservoir of entomopathogenic fungi as it provides shelter for insect fungal diseases [15–16]. Therefore, isolating new and functional entomopathogenic fungal strains from the soil is an effective and meaningful strategy for pest control. However, it is difficult to discover new distinct species and strains of soil fungi because the human activities lead to frequent genetic communications of organisms. The Qinghai-Tibet Plateau and Gansu Corridor areas are geographically remote and less disturbed by human. They possess distinct geographic and climatic conditions with the special biological diversity of glaciers, alpine meadows, deserts, forests and wetlands. They contain alpine arid and semiarid climates, with drought, cold, strong winds and high levels of solar radiation. The distinctive plateau climate and geographical environment promote the formation of the special biological diversity in those areas [17–18]. The Gansu Corridor is the desert oasis connecting central China and Xinjiang. It is also the pathway of the ancient Silk Road. Under its dry environmental conditions, many distinct crops such as sunflower, traditional Chinese medicinal plants, hops, etc., are planted [19–20]. Extreme environments benefit these districts separated from other areas. This means that some new fungal strains capable of whitefly biocontrol may exist in the soil of these areas.

To investigate the fungal entomopathogens that affect whiteflies in the soil of these two districts, the authors collected soil samples from south Tibet (east to Nyingchi and west to Xigaze), north Qinghai (south to the Gonghe region of Qinghai lake and north to the Jinyangling region of the Qilian Mountains) and the Gansu Corridor during 2013 and 2014 and compared the fungal strains between the two regions. It was predicted that the findings of this study could provide new insight into mycoinsecticides and the biocontrol of the whitefly pest.

## Materials and Methods

### Soil sample collection

**Fig 1:**
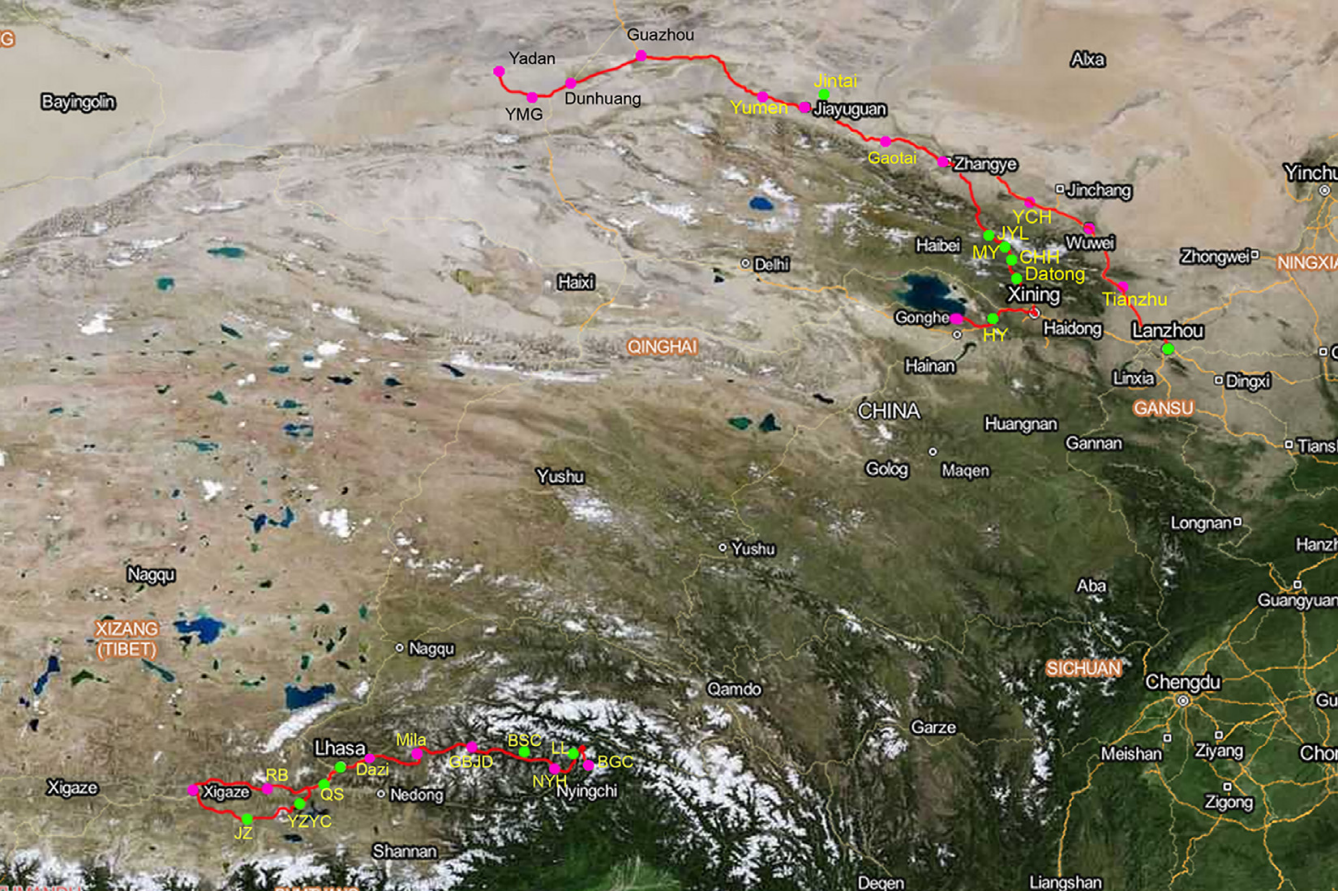
Map of the soil sample collection sites. Fungi were successfully isolated from locations labeled in green. Fungi could not be isolated from areas labeled in pink. The map was downloaded from“Map World of Public Platform of National Geographic Information of China (http://www.tianditu.com/). The south Tibet line: Xigaze, RB (Renbu), JZ (Jiangzhi, Ggangze), YZYC (Yangzhuoyongcuo, Langkazi,), QS (Qushui), Lhasa, Dazi, Mila (Mila Mountain, Mozhugongka), GBJD (Gongbujiangda), BSC (Basongcuo, Nyingchi), NYH (Niyang river, Nyingchi), LL (LuLang, Nyingchi), BGC (Brahmaputra grand canyon, Nyingchi). The Gansu corridor line: HY (Huangyuan, Qinghai), Datong (Datong, Qinghai), CHH (Chahanhe, Qinghai), MY (Menyuan, Qinghai), JYL (Jingyangling, Qinghai), Lanzhou (Yongdeng, Gansu), Jintai (Jintai, Gansu), Qilian (Qilian, Qinghai), Gonghe (Gonghe, Qinghai), Tianzhu (Tianzhu, Gansu), Wuwei (Wuwei, Gansu), YCH (Yongchang,Gansu), Zhangye (Zhangye, Gansu), Jiayuguan (Jiayuguan, Gansu), Yumen (Yumen, Gansu), Guazhou (Guazhou, Gansu), Dunhuang (Dunhuang, Gansu), YMG ((Yumenguan, Dunhuang, Gansu), Yadan (Dunhuang, Gansu).

The soil samples were collected from the locations covered with varying types of vegetation including forest, bush, grassland, thin grassland and desert in south Tibet (east to Nyingchi, west to Xigaze), north Qinghai (south to Gonghe of Qinghai lake, north to Jinyangling of Qilian Mountains) and the Gansu Corridor of China during 2013 and 2014 (Fig 1). From each location, approximately 200 g of soil 10 cm beneath the ground was collected along with samples from three randomly selected sites. Each sample was stored in a plastic bag at 4°Cfor further use.

### Isolation of fungal species from the soil samples

To isolate fungal strains, firstly the soil sample was pulverized and mixed. After mesh screening, 10 g of soil was collected and placed in a sterilized flask with 90 mL 0.1% Tween-80 solution, then agitated for 15 minutes to form a suspension. Two milliliters of the suspension was transferred to a clean flask containing 8 mL 0.05% Tween-80, and the mixture was shaken adequately. The final 0.1 mL suspension was inoculated onto a selective medium (1000 mL PDA, sterilized and cooled to 50°C, then augmented with 0.2 g actinone, 0.2 g alficetin and 0.0133 g rose-bengal)[21] and was spread homogeneously with a glass rod. Each soil sample was inoculated onto three plates. The plates were cultured at 25°C for 5 to 7 days. Then, the suspected fungal plaques were transferred onto PDA plates and cultured as above. Isolating the fungus in the plates to the PDA slants, the fungal strains were preserved for bioassay and classification.

### Identification of fungal species and analysis of genetic homology

The identification of fungal isolates was based on the morphological characteristics and genetic homology of the rDNA-ITS genes. The isolated slants were transferred to PDA plates and cultured for 3–5 days at 25°C. Then, a computer coupled optical microscope equipped with a digital camera (MC-D500U, Phenix, Jiangxi) was employed to carry on the morphological analysis.

The total DNA of a fungal strain was extracted using a DNA extraction kit (DP3112, BioTeke, Beijing). The fungal ITS region was amplified using the primers ITS1 and ITS4 [22]. The standard PCR cycling protocol used here included the following steps: an rDNA-ITS amplification procedure consisting of pre-degeneration at 94°C for 3 min, degeneration at 94°C for 30 s, annealing at 55°C for 30 s, and extension at 72°C for 1 min; this protocol was repeated for 35 cycles with a 72°Cextension for 10 min. The following mixture was then subjected to PCR in a reaction system (50μL): 22 μL ddH_2_O, 25μL 2×Power Tag PCR MasterMix (PR1701, Bio Teke, Beijing), 1 μL ITS1 primer 1, 1 μL ITS4 primer 1, and 1μl DNA template. Finally, the resulting PCR products were subjected to electrophoresis on a 1% agarose gel using Gold view I dye (3–10μL were loaded and run at 100 volts and 220 MA). The PCR products were visualized with UV illumination and photographed (Tanon-1600, Tanon, Shanghai) and sequenced by Sangon Biotech on an ABI-PRISM3730 automated sequencer (Applied Biosystems, USA). The obtained rDNA-ITS sequences were analyzed and compared with similar sequences through the BLAST on GenBank of NCBI and MEGA 5.0 software (IGE Biotechnology, Guangzhou).

### Bioassay of the fungal strains on the B-biotype whitefly

Firstly, fungal conidia suspensions were prepared. Conidial spores were collected from the PDA plates and suspended with sterilized 0.02% Tween-80 solution and calibrated to a stock of 1.0×10^8^ spores/mL for further use. Different working solutions (e.g. 9.0×10^7^, 3.0×10^7^, 5.0×10^7^, 1.0×10^7^, 5.0×10^6^, 1.0×10^6^ and 5.0×10^5^ spores/mL) prepared by diluting the stock with Tween-80 solution were used for the bioassays.

The population of B-biotype whiteflies used in this study was a greenhouse population reared for more than 20 generations. *Hibiscus rosa-sinensis* was selected to feed the insects. During the bioassay, adult whiteflies were moved onto the pot-planted *H*. *rosa-sinensis* for 24 hours to lay eggs. Subsequently, the pot plants with eggs were cultured in an incubator at 25°C, RH 70% under a photoperiod of 12 L: 12D.The 2^nd^ instar nymphs were used for the bioassay.

The leaf immersion method (China standard NY/T 1154.1 4–2008) was used to set up the bioassay. In brief, *H*. *rosa-sinensis* leaves with 2^nd^ instars’ whitefly nymphs were dipped into conidial working solutions for 20 s. After dryness, the leaves were cultured under the above conditions. The pest's numbers were surveyed each 24 h after treatment. The nymphs of *B*. *tabaci* were considered to be diseased when they lost their normal yellow-green colour, turgidity and smooth cuticle structure, and subsequently mildew grown[23]. The 0.02% tween-80 solution was used as a control group. The experiment was replicated three times.

### Statistical analysis

The calibrated accumulative mortality rate of the whitefly was calculated according to Abbott’s equation[24]. The LC50, the concentration lethal to half of any given species over a certain time, was calculated for each of the 7 fungal suspensions. The lethal time of 50% insects (LT50) was also determined. The LC_50_ and LT_50_ values were evaluated by means of probit analysis[25] employing the statistical software SPSS 17.0 (SPSS Inc., Chicago, IL, USA).

### Ethics Statement

There were no specific permissions required for these locations/activities. Because that the soil samples collected from locations covered with varying types of vegetation including forest, bush, grassland, thin grassland and desert in south Tibet, north Qinghai and the Gansu Corridor of China, were all belonging to the public property and allowed by the local land administrative departments of China government.

## Results

### Isolation and identification of entomogenous fungi in soil

In the 32 soil samples, 19 fungal strains were isolated from 11 soil samples (Fig 1, Table 1). The average isolation rate in south Tibet was 46.15%, while that of the Qinghai and Gansu Corridor was only 36.84%. Fungi had the highest frequency in the locations with good vegetation, including forest-, grass- and bush-covered regions. More specifically, fungal strains were isolated from all forest- and bush-covered regions and from 8 of the 13 grass-covered locations. However, no fungal strains were isolated from the soil collected from desert and thin grass regions (S1 Table).

**Table 1:** The 19 fungal strains isolated from the soil of the Qinghai-Tibet plateau and Gansu Corridor.

Location of soil sample collection		Fungal strain	
Name	Vegetation	Name	Species
Lhasa	Grass	MaTS01	*M*. *anisopliae* var. *anisopliae*
		IfTS08	*I*. *fumosorosea*
		BbTS02	*B*. *bassiana*
QS	Grass	PlTS02	*P*. *lilacinus*
JZ	Grass	MaTS02	*M*. *anisopliae* var. *anisopliae*
		BbTS01	*B*. *bassiana*
LL	Forest	MaTS03	*M*. *anisopliae* var. *anisopliae*
BSC	Forest	MaTS04	*M*. *anisopliae* var. *anisopliae*
YZYC	Grass	MaTS05	*M*. *anisopliae* var. *anisopliae*
HY	Grass	IfTS02	*I*. *fumosorosea*
Datong	Grass	IfTS05	*I*. *fumosorosea*
CHH	Forest	IfTS04	*I*. *fumosorosea*
MY	Grass	IfTS01	*I*. *fumosorosea*
JYL	Grass	IfTS03	*I*. *fumosorosea*
Lanzhou	Bush	AuTS01	*A*. *ustus*
		AuTS02	*A*. *ustus*
		PlTS01	*P*. *lilacinus*
		LpTS01	*L*. *psalliotae*
Jintai	Forest	BbTS03	*B*. *bassiana*

The full names of abbreviated regions were listed in the Fig 1 captions. The special information of 32 soil samples isolated from different vegetation types in Qinghai-Tibet plateau and Gansu Corridor were revealed in the S1 Table.

Based on the mophological and ITS sequences (S2 and S3 Tables, S1 Fig), they were identified as 6 species. Among them, *Isaria fumosorosea* with 6 isolated strains and an isolation rate of 31.58% was the most commonly isolated species. The second most common species was *Metarhizium anisopliae*, with 5 strains and a 26.31% isolation rate. Other identified species included *Beauveria bassiana* with 3 strains, *Aspergillus ustus* with 2 strains, *Paecilomyces lilacinus* with 2 strains and *Lecanicillium psalliotae* with 1 strain.

**Fig 2:**
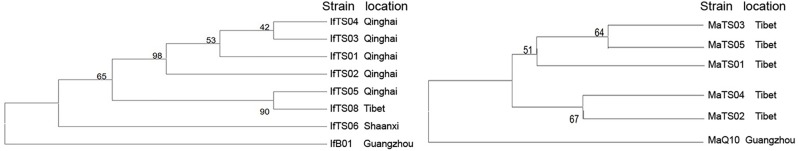
Phylogenetic trees of *I*. *fumosorosea* and *M*. *anisopliae* var. *anisopliae* strains based on the rDNA-ITS sequences. Bootstrap values are based on is 1,000 replicates. Numbers shown above branches are bootstrap percentages for clades supported above the 50% level.

Furthermore, the six strains of *I*. *fumosorosea* and five strains of *M*. *anisopliae* var. *anisopliae* from Qinghai-Tibet Plateau area were compared with the same species strains from other locations based on the rDNA-ITS sequences analysis. The results indicated that a larger homology existed in the strains inside Qinghai-Tibet Plateau area, but there were greater genetic difference between the strains from Qinghai-Tibet Plateau area and the strains from other location strains (Fig 2). It was illustrated that Qinghai-Tibet Plateau area are indeed isolated from other districts and less disturbed by human.

### Bioactivity of the entomopathogenic fungi against the B-biotype whitefly

**Fig 3:**
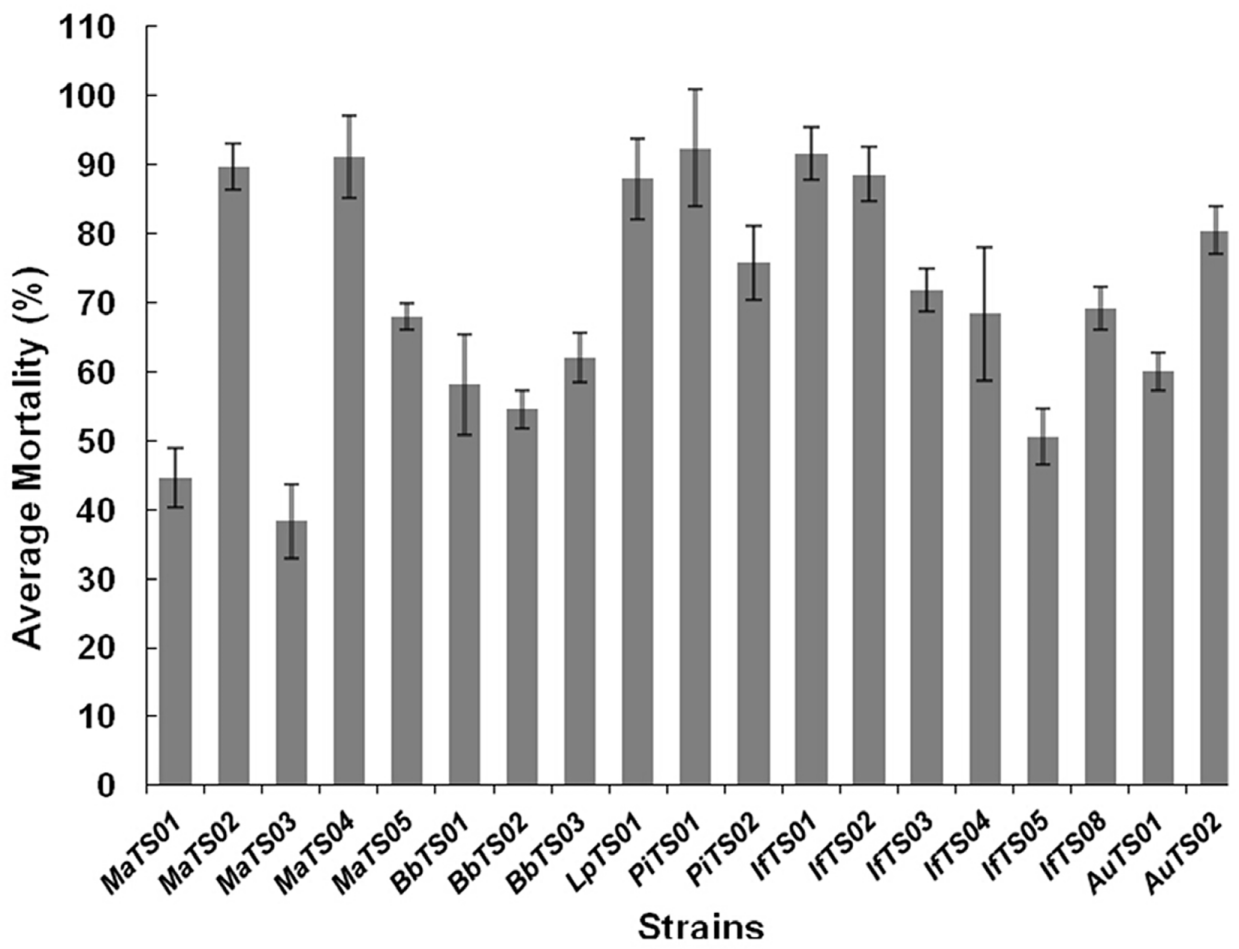
Mortality of 2^nd^ B-biotype whitefly nymphs infected by fungal isolates after 13 days of treatment. The letters on the columns indicated statistical significance (LSD, P<0.05).

**Fig 4:**
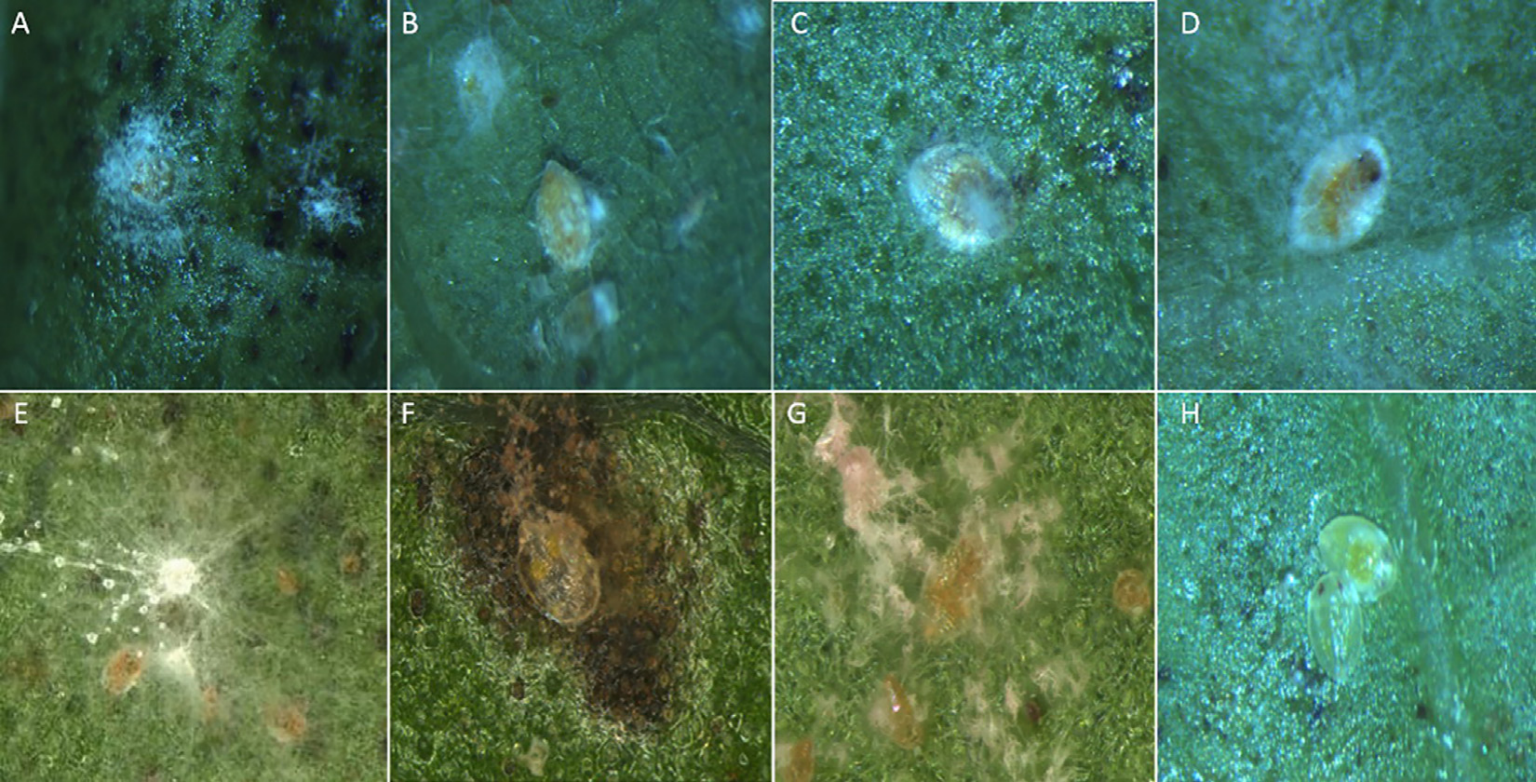
B-biotype whitefly nymphs were infected with fungal strains after one week of treatment. (A) IfTS01. (B) IfTS02. (C) MaTS02. (D) MaTS04. (E) LpTS01. (F) PlTS01. (G). AuTS02. (H) CK. The bodies of whiteflies larvae were thoroughly surrounded by mycelia and the colors were changed. It also observed that lots of conidia have generated under the microscope which shows that whiteflies larva have been dead for the infection of hypha.

**Fig 5:**
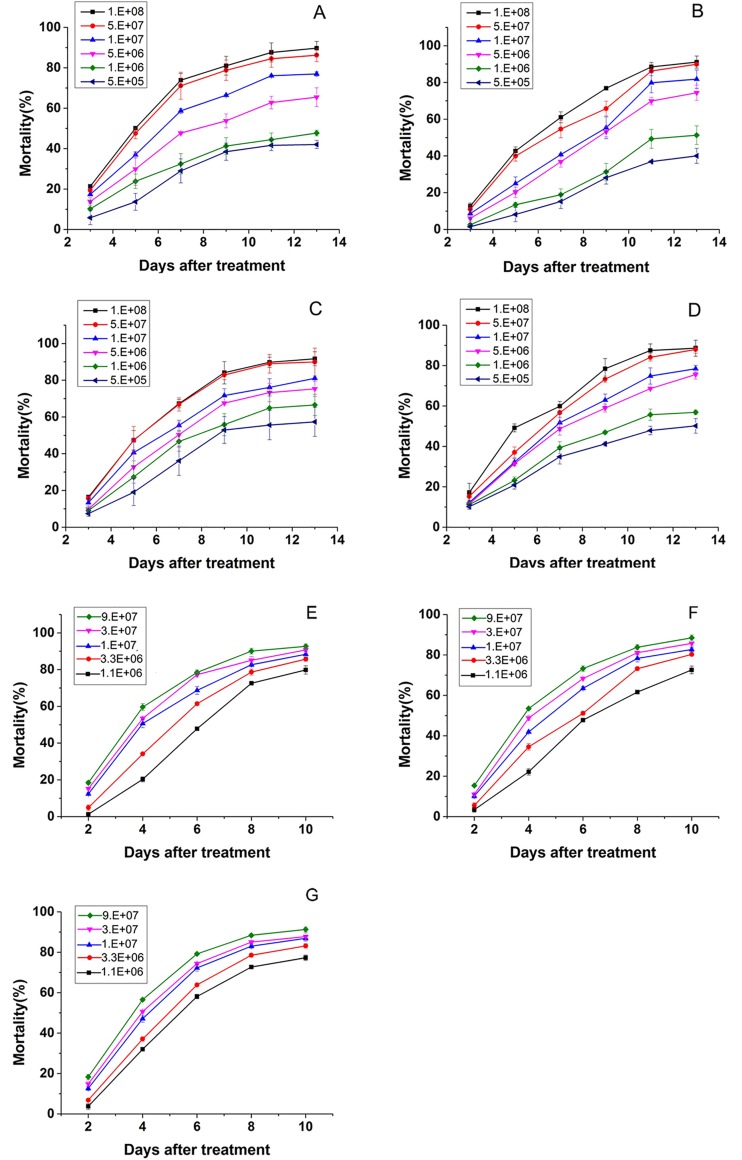
Cumulative corrected mortality of B-biotype whiteflies treated with the 7 fungal strains. (A)MaTS02. (B)MaTS04. (C)IfTS01. (D)IfTS02. (E)IfTS07. (F)PlTS01. (G)AuTS02. (H)LpTS01.

All of the total 19 entomopathogenic fungal strains produced substantial pathogenicities to B-biotype whiteflies with calibrated mortalities larger than 30% after 13 days of treatment, but the mortality rates varied among different strains (Fig 3). Based on the results, the 7 strains with high pathogenicity levels were selected for the virulence test. The further results indicated that the whiteflies started to grow mildew on their bodies approximately a week after treated by different strains (Fig 4).Overall, the mortality rates of the whiteflies were positively correlated with the dosages of fungal conidia concentrations and the length of time after the treatments (Fig 5).

The LC_50_ values ranged from 0.36×10^6^ spores/mL to 26.44×10^6^ spores/mL after treatment for 6–7 days (Table 2). Meanwhile, there were the LT_50_ values of 4.23–7.98 d (at 10×10^6^spores/mL concentration) (Table 3).The *L*. *psalliotae* strain LpTS01 showed the greatest virulence with a minimum LC_50_ value of 0.36×10^6^ spores/mL, while the *M*. *anisopliae* var. *anisopliae* strain MaTS04 had the lowest virulence with maximum LC_50_ values of 26.44×10^6^ spores/mL. Correspondingly, at a concentration of 10×10^6^ spores/mL, the LpTS01 strain had the minimum LT_50_ value of 4.23 d, and the MaTS04 strain had the maximum LT_50_ value of 7.98 d (Table 3). Furthermore, the strains PlTS01, AuTS02, IfTS01, MaTS02, and IfTS02 had gradually increasing LC_50_ values ranging from 1.66×10^6^ spores/mL to 9.00×10^6^ spores/mL and with corresponding LT_50_ values of 4.35–6.97 d.

**Table 2:** The equations of LC-p and LC_50_ values of fungal strains against B-biotype whiteflies.

Strain	Days after treatment (d)	Intercept	Slope	χ^2^	P	LC_50_(95%Confident Internal) (×10^6^ spores/mL)
**MaTS02**	7	-4.44	0.66	0.79	0.85	5.15(4.12–6.49)
**MaTS04**	7	-5.48	0.73	4.74	0.19	26.44(20.19–36.58)
**IfTS01**	7	-2.59	0.39	0.18	0.98	4.13(2.75–6.14)
**IfTS02**	7	-1.88	0.27	0.24	0.97	9.00(4.68–17.50)
**PlTS01**	6	-3.09	0.50	2.28	0.52	1.66(0.98–2.47)
**AuTS02**	6	-2.46	0.38	2.26	0.52	2.73(1.29–4.52)
**LpTS01**	6	-1.81	0.32	1.55	0.67	0.36(0.065–0.92)

**Table 3:** The equations of LT-p and LT_50_ values of fungal strains against B-biotype whiteflies.

Strain	Concentration	Intercept	Slope	χ^2^	P	LT_50_(95%Confident Internal) (d)
	(×10^6^ spores/mL)					
**MaTS02**	10	-2.26	2.80	5.67	0.34	6.40(6.10–6.71)
	5	-2.25	2.49	4.93	0.42	8.06(7.64–8.52)
**MaTS04**	10	-2.56	2.84	0.20	0.98	7.98(7.51–8.54)
	5	-3.49	3.84	6.14	0.19	8.11(7.85–8.93)
**IfTS01**	10	-2.27	2.99	4.93	0.30	5.73(5.45–6.02)
	5	-2.47	2.96	4.00	0.55	6.85(6.51–7.21)
**IfTS02**	10	-2.64	0.16	1.23	0.87	6.97(6.62–7.33)
	5	-2.55	0.20	0.41	0.98	7.40(6.90–7.93)
**PlTS01**	10	-2.23	3.49	0.57	0.90	4.35(4.12–4.59)
	3.3	-2.74	3.86	0.21	0.98	5.11(4.84–5.38)
**AuTS02**	10	-2.16	3.19	2.56	0.46	4.76(4.52–5.00)
	3.3	-2.59	3.48	3.49	0.32	5.56(5.27–5.85)
**LpTS01**	10	-2.09	3.34	4.71	0.19	4.23(4.04–4.42)
	3.3	-2.50	3.58	4.57	0.21	4.98(4.77–5.20)

## Discussion

The distribution of fungal entomopathogens is greatly influenced by many factors such as climate, geographical location, vegetation type, soil, organism and human activities, in addition to the characteristics of the fungi themselves. In our study, more fungal species and strains were found in Tibet and Qinghai than in Gansu. This might due to the larger variation of the geographical location and climatic type in the studied regions of Qinghai-Tibet Plateau area, since the temperature and humid of the former is higher than Gansu Corridor area. The better temperature and humid conditions determine the better vegetation structure. Therefore, most of Qinghai-Tibet Plateau areas were covered by forests and grasses, but the most of Gansu Corridor area was covered by deserts. In fact, in this research, more fungal strains were isolated in the soil covered with good vegetations, but no strains were discovered in deserts. It just suggests that the ground vegetation determines whether fungal entomopathogens exist in the soil. As it is known, there is a life cycle during which entomogenous fungal spores in the soil infect the host insects under suitable conditions and, after finishing their pathogenic progress, the new spores will leave the insect cadavers and enter the soil, where they will be stored until the next infection stage [26]. Therefore, in conditions where there is robust general biodiversity, there is also robust biodiversity in the entomogenous fungi species presenting in the soil.

In this research, *I*. *fumosorosea* was found to be the dominant isolated fungal species distributed in Tibet and Qinghai. This is likely because *I*. *fumosorosea* is actually a species complex, with a rich biodiversity and many host insects throughout the world [27]. The other rich species is *M*. *anisopliae* var. *anisopliae*. However, this species was only isolated in Tibet instead of Qinghai and Gansu. This corresponds well with the previous study in Sun *et*.*al* showing that *M*. *anisopliae*var. *anisopliae*was most frequently found in warmer regions[28], since geographically the climate of Tibet warmer than the Qinghai and Gansu. What’s more, by comparing the ITS genetic homology of the *I*. *fumosorosea* and *M*. *anisopliae* var. *anisopliae* strains from this research with other places in China, a lower level of homology was identified between them. It is verified that the Qinghai-Tibet Plateau area was less disturbed by human, and the fungi in this place exchanged less with other regional species.

To date, there are no reports about B-biotype whiteflies occurrence and epidemic in Qinghai-Tibet area. However, many fungal trains of whitefly's pathogen were isolated in this research. We think the one reason is that many entomogenous fungal species widely and commonly infects in various hosts including insects, mites and nematodes. It survives in different habits including soil as saprophyte and attacks different stages of insects. Another reason is maybe there are many the relative species of B-biotype whitefly, some local species or biotypes distributed in Qinghai-Tibet area.

According to the bioassay, seven strains with higher pathogenicity against biotype whitefly were found. The most interesting results were found for the dominant isolated species, *I*. *fumosorosea* and *M*. *anisopliae*var. *anisopliae*, although these were not the most effective strains for whitefly control. In contrast, *P*. *lilacinus*, *L*. *psalliotae* and *A*. *ustus*, which were isolated at lower frequencies, have higher virulence. Unlike *I*. *fumosorosea*widely used for whitefly biocontrol [11, 23] and *M*. *anisopliae* var. *anisopliae a*s a popular mycoinsecticide, *L*. *psalliotae*, *P*. *lilacinus* and *A*. *ustus* have rarely been reported to be used for whitefly control, although *L*. *psalliotae* and *P*. *lilacinus* have received much attention for nematode biocontrol [29–31]. In fact, *A*. *ustus* is an attractive species for researchers because of its human pathogen potential [32–33]. In this study, we observed that the strain AuTS02 of *A*. *ustus* usually causes plant necrosis (marked by the appearance of black spots) in addition to infecting whiteflies. This suggests that this fungus may produce toxins fatal to plants cells. Therefore, the strain AuTS02 of *A*. *ustus* is not a good choice for a pest bio-control agent.

In conclusion, 19 strains of 6 fungal entomopathogens were isolated from soil samples of 32 locations in the Tibet-Qinghai Plateau and Gansu Corridor. However, all strains were only isolated from the soil covered by forests, bush and grass, while entomogenous fungi could not be found in the desert soil and places with thin grass vegetation. These results suggest that where there is good vegetation, there is also good biodiversity of entomogenous soil-dwelling fungi. In the present research, *I*. *fumosorosea* and *M*. *anisopliae* var. *anisopliae* were the major isolated species. The ITS genetic homology indicates that the two species in the Qinghai-Tibet Plateau are different from the strains of other districts. It is verified that the Qinghai-Tibet Plateau area was less disturbed by human, and the fungi in this place exchanged less with other regional species. All of the strains had some pathogenicity to whitefly, but a few strains of *P*. *lilacinus*, *L*. *psalliotae*,*A*. *ustus*, *I*. *fumosorosea* and *M*. *anisopliae* var. *anisopliae* had more significant levels of virulence. In particular, the *P*. *lilacinus* strain PlTS01 and *L*. *psalliotae* strain LpTS01 were shown to the greatest potential as B-biotype whitefly bio-control agents.

## Supporting Information

S1 Fig The morphological characteristics of 7 fungal strainswith higher virulence. (A)AuTS02.(B)LpTS01.(C)MaTS04.(D) IfTS02.(E) MaTS02.(F)IfTS01.(G) PiTS01.Bar = 10 μm.
(TIF)

S1 Table Strains isolated from the soil of the Qinghai-Tibet plateau and Gansu Corridor. 19 fungal strains belonging to 6 species isolated from 32 soil samples of various vegetation types.
(PDF)

S2 Table The ITS sequences of fungal strains. (PDF)

S3 Table The size of sporogenous structure of 7 strains with higher virulence. The data gives the size scope of each feather to compare with the corresponding fungal species.
(PDF)
