# Supplementary figures and images for: Isolation and Classification of Fungal Whitefly Entomopathogens from Soils of Qinghai-Tibet Plateau and Gansu Corridor in China
Source: PLoS One. 2016 May 26;11(5):e0156087. doi: 10.1371/journal.pone.0156087 (PMC4881913; doi:10.1371/journal.pone.0156087)

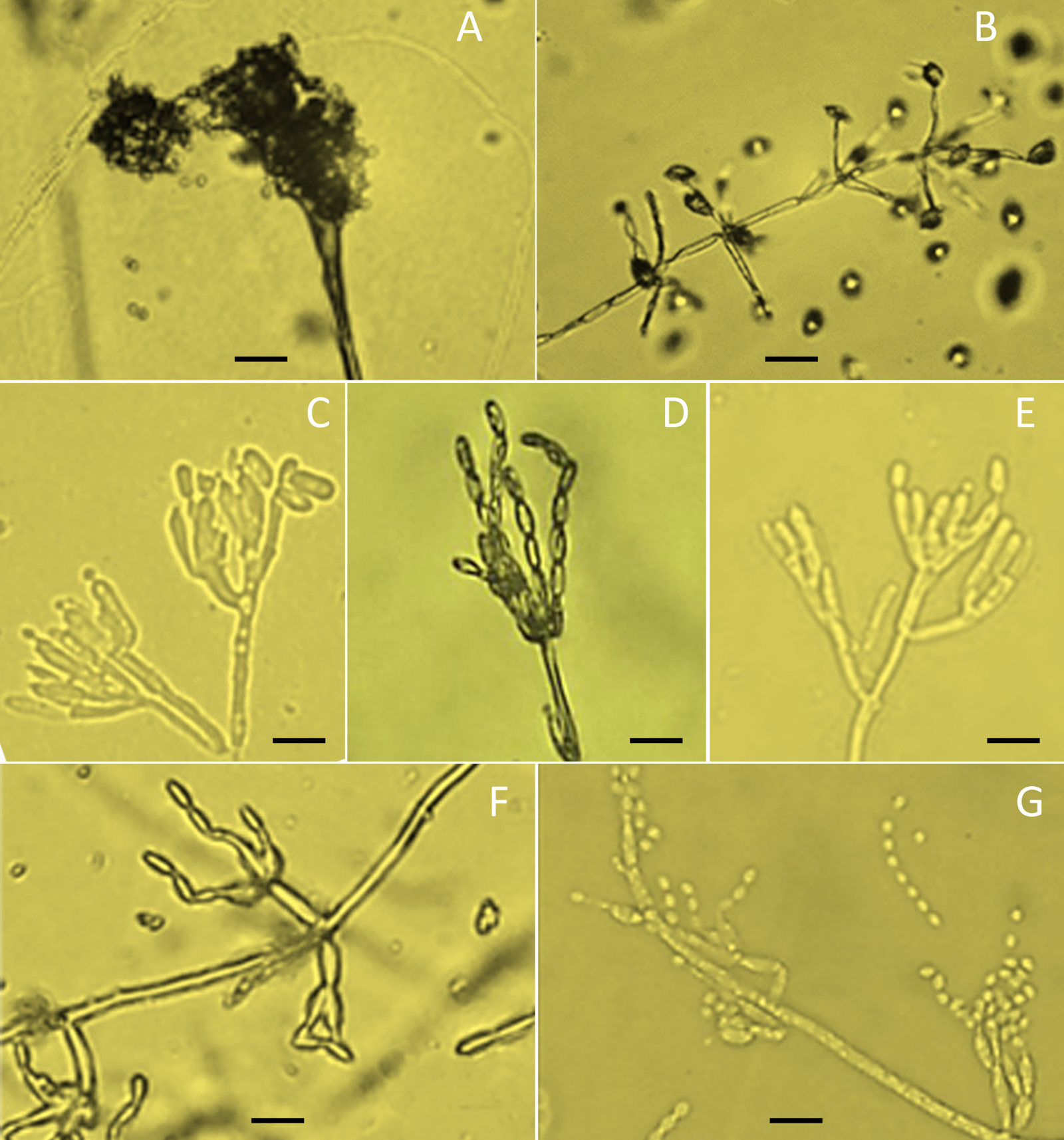

Supplement: S1 Fig — (A)AuTS02.(B)LpTS01.(C)MaTS04.(D) IfTS02.(E) MaTS02.(F)IfTS01.(G) PiTS01.Bar = 10 μm. (TIF) [file pone.0156087.s001.tif]
