# Supplementary material for: Isolation and Classification of Fungal Whitefly Entomopathogens from Soils of Qinghai-Tibet Plateau and Gansu Corridor in China
Source: PLoS One. 2016 May 26;11(5):e0156087. doi: 10.1371/journal.pone.0156087 (PMC4881913; doi:10.1371/journal.pone.0156087)
Supplement: S1 Table — 19 fungal strains belonging to 6 species isolated from 32 soil samples of various vegetation types. (PDF) [file pone.0156087.s002.pdf]

S1 Table. Strains isolated from the soil of the Qinghai-Tibet plateau and Gansu Corridor.

NO.	Location of soil sample collection			Fungal strain	
	**Name**	**Latitude and longitude**	**Vegetation**	**Name**	**Species**
1	Lhasa (Lhasa, Tibet)	E91.226, N29.665	Grass	MaTS01	*M. anisopliae* var. *anisopliae*
				IfTS08	*I. fumosorosea*
				BbTS02	*B. bassiana*
2	QS (Qushui, Tibet)	E90.665, N29.322	Grass	PlTS02	*P. lilacinus*
3	JZ (Jiangzhi, Ggangze, Tibet)	E90.171, N28.900	Grass	MaTS02	*M. anisopliae* var. *anisopliae*
				BbTS01	*B. bassiana*
4	LL (LuLang, Nyingchi, Tibet)	E94.814, N29.948	Forest	MaTS03	*M. anisopliae* var. *anisopliae*
5	BSC (Basongcuo, Nyingchi, Tibet)	E93.955, N30.001	Forest	MaTS04	*M. anisopliae* var. *anisopliae*
6	YZYC (Yangzhuoyongcuo, Langkazi, Tibet)	E90.411, N29.978	Grass	MaTS05	*M. anisopliae* var. *anisopliae*
7	Xigaze (Xigaze, Tibet)	E88.928, N29.320	Thin grass	___	___
8	RB (Renbu, Tibet)	E89.983, N29.344	Thin grass	___	___
9	Dazi (Dazi, Tibet)	E91.307, N29.669	Thin grass	___	___
10	Mila (Mila Mountain, Mozhugongka, Tibet)	E92.356, N29.832	Thin grass	___	___
11	GBJD (Gongbujiangda, Tibet)	E93.161, N29.941	Thin grass	___	___
12	NYH (Niyang river, Nyingchi, Tibet)	E94.011, N29.759	Grass	___	___
13	BGC (Brahmaputra grand canyon, Nyingchi, Tibet)	E94.892, N29.522	Thin grass	___	___
14	HY (Huangyuan, Qinghai)	E101.201, N36.568	Grass	IfTS02	*I. fumosorosea*
15	Datong (Datong, Qinghai)	E101.671, N36.813	Grass	IfTS05	*I. fumosorosea*
16	CHH (Datong, Qinghai)	E101.554, N37.253	Forest	IfTS04	*I. fumosorosea*
17	MY (Menyuan, Qinghai)	E101.464, N37.391	Grass	IfTS01	*I. fumosorosea*
18	JYL (Menyuan, Qinghai)	E101.420, N37.410	Grass	IfTS03	*I. fumosorosea*
19	Lanzhou (Yongdeng, Gansu)	E103.621, N36.470	Bush	AuTS01	*A. ustus*
				AuTS02	*A. ustus*
				PlTS01	*P. lilacinus*
				LpTS01	*L. psalliotae*
20	Jintai (Jintai, Gansu)	E98.823, N40.051	Forest	BbTS03	*B. bassiana*

**21**	Qilian (Qilian, Qinghai)	E100.939, N37.984	Grass	___	___
**22**	Gonghe (Gonghe, Qinghai)	E100.678, N36.553	Grass	___	___
**23**	Tianzhu (Tianzhu, Gansu)	E103.171, N36.961	Thin grass	___	___
**24**	Wuwei (Wuwei, Gansu)	E102.661, N37.930	Thin grass	___	___
**25**	YCH (Yongchang,Gansu)	E101.910, N38.281	Thin grass	___	___
**26**	Zhangye (Zhangye, Gansu)	E100.092, N39.012	Thin grass	___	___
**27**	Jiayuguan (Jiayuguan, Gansu)	E98.411, N39.923	Thin grass	___	___
**28**	Yumen (Yumen, Gansu)	E97.415, N40.030	Grass	___	___
**29**	Guazhou (Guazhou, Gansu)	E95.912, N40.560	Grass	___	___
**30**	Dunhuang (Dunhuang, Gansu)	E94.701, N40.090	Thin grass	___	___
**31**	YMG (Yumenguan, Dunhuang, Gansu)	E93.875, N40.357	Thin grass	___	___
**32**	Yadan (Dunhuang, Gansu)	E93.195, N40.442	Thin grass	___	___
