# Supplementary material for: Isolation and Classification of Fungal Whitefly Entomopathogens from Soils of Qinghai-Tibet Plateau and Gansu Corridor in China
Source: PLoS One. 2016 May 26;11(5):e0156087. doi: 10.1371/journal.pone.0156087 (PMC4881913; doi:10.1371/journal.pone.0156087)
Supplement: S2 Table — (PDF) [file pone.0156087.s003.pdf]

S2 Table. The ITS sequences of fungal strains.

Strain	Species	Gene bank access No.	ITS seguence
MaTS01	*M.anisopliae* var.*anisopliae*		gtggctcgacttcactccacccctgtgattatacctttaattgttgcttcggcgggacttcgcgcccgccggggacccaaaccttctgaattttttaataagtatcttctgagtggttaaaaaaaaatgaatcaaaactttcaacaacggatctcttggttctggcatcgatgaagaacgcagcgaaatgcgataagtaatgtgaattgcagaattcagtgaatcatcgaatctttgaacgcacattgcgcccgtcagtattctggcgggcatgcctgttcgagcgtcattacgcccctcaagtcccctgcggacttggtgttggggatcggcgaggctggttttccagcacagccgtcccttaaattaattggcggtctcgccgtggccctcctctgcgcagtagtaaagcactcgcaacaggagcccggcgcggtccactgccgtaaaacccccaactttttatagttgacctcgaatcaggtaggactacccgctgaacttaagcatatcaa
IfTS08	*I.fumosorosea*		gggggatcggacttcactcccaacccactgtgaaccttacctcagttgcctcggcgggaacgccccggccgccggcccccgcgccggcgccggacccaggcgcccgccgcagggaccccaaactctcttgcattacgcccagcgggcggaatttcttctctgagttgcacaagcaaaaacaaatgaatcaaaactttcaacaacggatctcttggttctggcatcgatgaagaacgcagcgaaatgcgataagtaatgtgaattgcagaattcagtgaatcatcgaatcttgaacgcacattgcgcccgccagcattctggcgggcatgcctgttcgagcgtcatttcaaccctcgagcccccccgggggcctcggtgttggggggacggcacaccagccgcccccgaaatgcagtggcgaccccgccgcagcctcccctgcgtagtagcacacacctcgcaccggagcgcggaggcggtcacgccgtaaaacgcccaactttcttagagttgacctcggatCaggtaggaatacccgctgaacttaagcatatcaataagcggaggaa
MaTS02	*M.anisopliae* var.*anisopliae*	KX057378	ggggatcgacttcactcccacccctgtgattatacctttaattgttgcttcggcgggacttcgcgcccgccggggacccaaaccttctgaattttttaataagtatcttctgagtggttaaaaaaatgaatcaaaactttcaacaacggatctcttggttctggcatcgatgaagaacgcagcgaaatgcgataagtaatgtgaattgcagaattcagtgaatcatcgaatctttgaacgcacattgcgcccgtcagtattctggcgggcatgcctgttcgagcgtcattacgcccctcaagtcccctgtggacttggtgttggggatcggcgaggctggttttccagcacagccgtcccttaaattaattggcggtctcgccgtggccctcctctgcgcagtagtaaaacactcgcaacaggagcccggcgcggtccactgccgtaaaacccccaactttttatagttgacctcgaatcaggtaggactacccgctgaacttaagcatatcaaaagccggaggaa
BbTS01	*B. bassiana*		ggggatcggagcttcactcctacccttctgtgacctacctatcgttgcttcggcggactcgccccagcccggacgcggactggaccagcggcccgccggggacctcaaactcttgtattccagcatcttctgaatacgccgcaaggcaaaacaaatgaatcaaaactttcaacaacggatctcttggctctggcatcgatgaagaacgcagcgaaacgcgataagtaatgtgaattgcagaatccagtgaatcatcgaatctttgaacgcacattgcgcccgccagcattctggcgggcatgcctgttcgagcgtcatttcaaccctcgacctccccttgggggggtcggcgttggggaccggcagcacaccgccggccctgaaatggagtggcggcccgtccgcggcgacctctgcgcagtaatacagctcgcaccggaaccccgacgcggccacgccgtaaaacacccaacttctgaacgttgacctcgaatcaggtaggactacccgctgaacttaagcatatcaaaagccggaggaa
MaTS03	*M.anisopliae* var.*anisopliae*		gtggcctcgactacactcccacccctgtgattatacctttaattgttgcttcggcgggacttcgcgcccgccggggacccaaaccttctgaattttttaataagtatcttctgagtggttaaaaaaatgaatcaaaactttcaacaacggatctcttggttctggcatcgatgaagaacgcagcgaaatgcgataagtaatgtgaattgcagaattcagtgaatcatcgaatctttgaacgcacattgcgcccgtcagtattctggcgggcatgcctgttcgagcgtcattacgcccctcaagtcccctgtggacttggtgttggggatcggcgaggctggttttccagcacagccgtcccttaaattaattggcggtctcgccgtggccctcctctgcgcagtagtaaaacactcgcaacaggagcccggcgcggtccactgccgtaaaacccccaactttttatagttgacctcgaatcaggtaggactacccgctgaacttaagcatatcta
MaTS04	*M.anisopliae* var.*anisopliae*	KX057377	ggggaatcgacttccactcccacccctgtgattatacctttaattgttgcttcggcgggacttcgcgcccgccggggacccaaaccttctgaattttttaataagtatcttctgagtggttaaaaaaatgaatcaaaactttcaacaacggatctcttggttctggcatcgatgaagaacgcagcgaaatgcgataagtaatgtgaattgcagaattcagtgaatcatcgaatctttgaacgcacattgcgcccgtcagtattctggcgggcatgcctgttcgagcgtcattacgcccctcaagtcccctgtggacttggtgttggggatcggcgaggctggttttccagcacagccgtcccttaaattaattggcggtctcgccgtggccctcctctgcgcagtagtaaaacactcgcaacaggagcccggcgcggtccactgccgtaaaacccccaactttttatagttgacctcgaatcaggtaggactacccgctgaacttaagcatatcaaaagccggaggaa
MaTS05	*M.anisopliae* var.*anisopliae*		ggctcgacacactcccacccctgtgattatacctttaattgttgcttcggcgggacttcgcgcccgccggggacccaaaccttctgaattttttaataagtatcttctgagtggttaaaaaaatgaatcaaaactttcaacaacggatctcttggttctggcatcgatgaagaacgcagcgaaatgcgataagtaatgtgaattgcagaattcagtgaatcatcgaatctttgaacgcacattgcgcccgtcagtattctggcgggcatgcctgttcgagcgtcattacgcccctcaagtccccctgtggacttggtgttggggatcggcgaggctggttttccagcacagccgtcccttaaattaattggcggtctcgccgtggccctcctctgcgcagtagtaaaacactcgcaacaggagcccggcgcggtccactgccgtaaaa

			cccccccaactttttatagttgacctcgaatcaggtaggactacccgctgaacttaagcatatcaaa
IfTS02	*I.fumosorosea*	KX057373	gggggtacgagcttttcactccctaaccctttgtgacatacctatcgttgcttcggcggactcgccccggcgtccggacggccctgcgccgcccgcgacccggacccaggcggccgccggagacccacaaattctgtttctatcagtctttctgaatccgccgcaaggcaaaacaaatgaatcaaaactttcaacaacggatctcttggttctggcatcgatgaagaacgcagcgaaatgcgataagtaatgtgaattgcagaattcagtgaatcatcgaatctttgaacgcacattgcgcccgccagcattctggcgggcatgcctgttcgagcgtcatttcaaccctcgacacccccttcgggggagtcggcgttggggaccggcagcataccgccggccccgaaatacagtggcggcccgtccgcggcgacctctgcgtagtactccaacgcgcaccgggaacccgacgcggccacgccgtaaaacacccaacttctgaacgttgacctcggatcaggtaggactacccgctgaacttaagcatatcaaaagccgggaggaa
IfTS05	*I.fumosorosea*		gattcggagctttcactccaaacccactgtgaacatataccatttgtttattcgttgcctcggcgggttctacccccctggagacagggcggccagcccccgccggcgtaacaccaaaaaccctgaatgtgtacccgttacacggcagtattactctgagtcacatcattttaaatgaatcaaaactttcaacaacggatctcttggttctggcatcgatgaagaacgcagcgaaatgcgataagtaatgtgaattgcagaattcagtgaatcatcgaatctttgaacgcacattgcgcccgccagtattctggcgggcatgcctgttcgagcgtcatttcaaccctcaggtccccctttttcggagggggagacaccctggtgttgggggacggcatcctggcccgctgtctcacagtgtgccacgccgcccccgaaatgaattggcggcctcgtcgcgtgccacccctgcgtagtagcacaacctcgcaacgggagcccgacgcggccactgccgtaaaacgcccaacttttaccgagttgacctcgaatcaggtaggaatacccgctgaacttaagcatatcaataagcggaggaa
IfTS04	*I.fumosorosea*		ggggatacgggctttttcactccctaaccctttgtgacatacctatcgttgcttcggcggactcgccccggcgtccggacggccctgcgccgcccgcgacccggacccaggcggccgccggagacccacaaattctgtttctatcagtctttctgaatccgccgcaaggcaaaacaaatgaatcaaaactttcaacaacggatctcttggttctggcatcgatgaagaacgcagcgaaatgcgataagtaatgtgaattgcagaattcagtgaatcatcgaatctttgaacgcacattgcgcccgccagcattctggcgggcatgcctgttcgagcgtcatttcaaccctcgacacccccttcgggggagtcggcgttggggaccggcagcataccgccggccccgaaatacagtggcggcccgtccgcggcgacctctgcgtagtactccaacgcgcaccgggaacccgacgcggccacgccgtaaaacacccaacttctgaacgttgacctcggatcaggtaggactacccgctgaacttaagcatatcaaaagcccggaggaaa
IfTS01	*I.fumosorosea*	KX057375	gggggctacggggcttttcactccctaaccctttgtgacatacctatcgttgcttcggcggactcgccccggcgtccggacggccctgcgccgcccgcgacccggacccaggcggccgccggagacccacaaattctgtttctatcagtctttctgaatccgccgcaaggcaaaacaaatgaatcaaaactttcaacaacggatctcttggttctggcatcgatgaagaacgcagcgaaatgcgataagtaatgtgaattgcagaattcagtgaatcatcgaatctttgaacgcacattgcgcccgccagcattctggcgggcatgcctgttcgagcgtcatttcaaccctcgacacccccttcgggggagtcggcgttggggaccggcagcataccgccggccccgaaatacagtggcggcccgtccgcggcgacctctgcgtagtactccaacgcgcaccgggaacccgacgcggccacgccgtaaaacacccaacttctgaacgttgacctcggatcaggtaggactacccgctgaacttaagcatatcaaaagccggaggaa
IfTS03	*I.fumosorosea*		ggggattcggggcttttcactccctaaccctttgtgacatacctatcgttgcttcggcggactcgccccggcgtccggacggccctgcgccgcccgcgacccggacccaggcggccgccggagacccacaaattctgtttctatcagtctttctgaatccgccgcaaggcaaaacaaatgaatcaaaactttcaacaacggatctcttggttctggcatcgatgaagaacgcagcgaaatgcgataagtaatgtgaattgcagaattcagtgaatcatcgaatctttgaacgcacattgcgcccgccagcattctggcgggcatgcctgttcgagcgtcatttcaaccctcgacacccccttcgggggagtcggcgttggggaccggcagcataccgccggccccgaaatacagtggcggcccgtccgcggcgacctctgcgtagtactccaacgcgcaccgggaacccgacgcggccacgccgtaaaacacccaacttctgaacgttgacctcggatcaggtaggactacccgctgaacttaagcatatcaaaagccggaggaa
AuTS01	*A. ustus*		ggggctccgagtcagtctgccccgggcaggcctaacctcccacccgtgaatacctgaccaacgttgcttcggcggtgcgcccccccgggggtagccgccggagaccacattgaacctcttgtctttagtgttgtctgagcttgatagcaaacctattaaaactttcaacaatggatctcttggttccggcatcgatgaagaacgcagcgaactgcgataagtaatgtgaattgcagaattcagtgaatcatcgagtctttgaacgcacattgcgccccctggcattccgggggggcatgcctgtccgagcgtcattgctgcccttcaagcccggcttgtgtgttgggtcgtcgtcccccccgggggacgggcccgaaaggcagcggcggcaccgcgtccggtcctcgagcgtatggggctttgtcacccgctcgattagggccggccgggcgccagccggcgtctccaaccttctattttaccaggttgacctcggatcaggtagggatacccgctgaacttaagcatatcaataagcggaggaa
AuTS02	*A. ustus*	KX057376	aggatacggtgcagtctgccccgggcaggcctaacctcccacccgtgaatacctgaccaacgttgcttcggcggtgcgcccccccgggggtagccgccggagaccacattgaacctcttgtctttagtgttgtctgagcttgatagcaaacctattaaaactttcaacaatggatctcttggttccggcatcgatgaagaacgcagcgaactgcgataagtaatgtgaattgcagaattcagtgaatcatcgagtctttgaacgcacattgcgccccctggcattccgggggggcatgcctgtccgagcgtcattgctgcccttcaagcccggcttgtgtgttgggtcgtcgtcccccccgggggacgggcccgaaaggcagcggcggcaccgcgtccggtcctcgagcgtatggggctttgtcacccgctcgattagggccggccgggcgccagccggcgtctccaaccttctattttaccaggttgacctcggatcaggtagggataccc

			gctgaacttaagcatatcaataagccggaggaa
PlTS01	*P. lilacinus*	KX057374	gataggggcttcactcccaacccactgtgaaccttacctcagttgcctcggcgggaacgccccggccgccggcccccgcgccggcgccggacccaggcgcccgccgcagggaccccaaactctcttgcattacgcccagcgggcggaatttcttctctgagttgcacaagcaaaaacaaatgaatcaaaactttcaacaacggatctcttggttctggcatcgatgaagaacgcagcgaaatgcgataagtaatgtgaattgcagaattcagtgaatcatcgaatctttgaacgcacattgcgcccgccagcattctggcgggcatgcctgttcgagcgtcatttcaaccctcgagcccccccgggggcctcggtgttgggggacggcacaccagccgcccccgaaatgcagtggcgaccccgccgcagcctcccctgcgtagtagcacacacctcgcaccggagcgcggaggcggtcacgccgtaaaacgcccaactttcttagagttgacctcggatcaggtaggaatacccgctgaacttaagcatatcaataagcggaggaa
LpTS01	*L. psalliotae*	KX057379	gggggaaggggctcactcccaacccttatgtgaacataccataatgttgcttcggcggactcgccccggcgtccggacggcctagcgccgcccgcggcccggacccaggcggccgccggagaccaccaaaactattttgtatcagcagtttttctgaatccgccgcaaggcaaaacaaatgaatcaaaactttcaacaacggatctcttggttctggcatcgatgaagaacgcagcgaaatgcgataagtaatgtgaattgcagaattcagtgaatcatcgaatctttgaacgcacattgcgcccgccagcattctggcgggcatgcctgttcgagcgtcatttcaaccctcgacttccctttggggaaatcggcgttggggactggcagcataccgccggccccgaaatggagtggcggcccgtccgcggcgacctctgcgtagtaatccaacctcgcaccggaaccccgacgtggccacgccgtaaaacaccccactttctgaacgttgacctcggatcaggtaggaatacccgctgaacttaagcatatcaaaagccggaggaa
