# Supplementary material for: Isolation and Classification of Fungal Whitefly Entomopathogens from Soils of Qinghai-Tibet Plateau and Gansu Corridor in China
Source: PLoS One. 2016 May 26;11(5):e0156087. doi: 10.1371/journal.pone.0156087 (PMC4881913; doi:10.1371/journal.pone.0156087)
Supplement: S3 Table — The data gives the size scope of each feather to compare with the corresponding fungal species. (PDF) [file pone.0156087.s004.pdf]

S4 Table. The size of sporogenous structure of 7 strains with higher virulence. The data gives the size scope of each feather to compare with the corresponding fungal species.

Strain	Conidia		Hyphae(μm)	Phialide(μm)	
	length (μm)	width (μm)	diameter (μm)	length (μm)	width (μm)
MaTS02	5.80-8.71	2.48-4.48	2.49-4.12	10.31-17.09	3.15-4.03
MaTS04	5.18-7.46	2.71-3.50	2.28-3.11	8.60-12.16	2.49-3.56
LpTS01	2.49-4.40	0.96-1.71	1.38-3.46	5.31-30.82	0.69-1.41
AuTS02	2.52-3.48	2.33-3.40	2.47-4.36	6.21-8.24	2.50-3.71
PlTS01	2.51-3.66	1.77-2.66	1.58-3.61	6.07-7.44	2.77-3.66
IfTS01	3.06-6.33	1.81-2.81	1.51-2.52	5.06-8.33	2.87-3.98
IfTS02	4.21-6.44	1.81-2.87	2.28-4.53	4.72-7.46	3.06-5.03
